# The Widening Field of the Dental Profession

**Published:** 1893-11

**Authors:** Frank W. Sage


### THE DENTAL REGISTER.

VOL. XLVII.] NOVEMBER, 1893. [No. 11.

### COMMUNICATIONS.

### The Widening Field of the Dental Profession.

BY FRANK W. SAGE, D.D S.

Read before the Cincinnati Odontological Society.

It is a curious and interesting fact that both before and since the recognition of dentistry as a specialty of medicine the chief expressions of dissent have come from the ranks of the profession so recognized. Up to the time of this official recognition, when on various occasions dentists had been invited provisionally to take part in medical conventions, some prominent men among the dentists expressed themselves in terms of disapproval, amounting almost to resentment. But when distinct recognition finally came this open opposition suddenly ceased, and dentists went about exchanging congratulations, not giving themselves any particular concern as to individual eligibility to the ranks of the older profession, under the conditions named in the resolution of recognition. It was enough to know that the door had been opened; dentists in some considerable numbers—not the largest perhaps, such as might have been drawn into dental conventions—flocked into the dental sections of the medical conventions, anxious to secure personal recognition. There is reason to suspect that the credentials of some of them were not very keenly scrutinized; at all events, it is not certain that any one was challenged as to his fitness, provided he could write the D.D.S. after his name. It was not brought to light that any committee of medical men had inquired and reported as to the standing of individual dental colleges, with a view to requiring conformity to the express conditions named in the resolution admitting their graduates to medical conferences. In short, it appeared that the medical profession was good-naturedly lenient in this particular matter, feeling probably that the dentists were ambitious, dead in earnest, and not likely to abuse their privileges.

In the first general convention of physicians and dentists held after this official recognition, it may be recalled that the dentists in their section all but ignored dentistry proper. Papers on skulls, facial tumors, physiognomy, oral surgery, and so on, were read and sparingly discussed by members who seemed to be over-awed by the momentous character of the occasion. It was amusing, almost ludicrous in its solemnity, and yet, in a sense, pitiful, in the constraint imposed upon the participants. The session was notable in one respect: with one or two exceptions the papers read were by men who bore the double title M.D., D.D.S.

All things considered the effect of this meeting was wholesome. It stirred and stimulated the dentists and brought them to realize that study and work were required of them individually if they were to sustain themselves in the position to which they had been assisted by the older sister profession. Meanwhile, during the lapse of years, since the first assembling of physicians and dentists in conventions, some dentists who have neither actually held aloof, nor actually participated in these conventions, have continued to doubt the expediency of this practical attempt to graft dentistry upon the parent stock. They have ceased to air their views and opinions in the dental journals, but there are indications that they still hold them, waveringly perhaps, because of inability to solve the many complex problems which are presented, from whatever standpoint they view the matter. The profession stands to-day in the anomalous attitude of having accepted, and yet not having accepted this invitation extended by the medical profession. While there has been no express declination from any quarter, there still prevails this feeling of indecision, such as might not unreasonably be looked for, when we consider the heterogeneous character of the body of the dental profession. Meanwhile we are witnessing the singular spectacle of one class of men in the profession—those who find themselves already qualified or able to qualify—flocking eagerly to the newly-erected standard; another class, more conservative and professing satisfaction with the highest standard previously erected by dentists, waiting in a non-committal attitude; and a third class, with a reputation based more expressly on mechanical ingenuity and operative skill, helplessly drifting in the current of popular opinion and wholly unable to surmise how and where they are to land.

But whatever may be the individual opinion of the question still being privately discussed, "Is Dentistry a Specialty of Medicine," the fact can no longer be ignored that a new era in dentistry is opening before us. Like other advance movements this impulse, looking to a higher standard of attainments for students of dentistry in our dental colleges, appears to be not directly the result of concerted effort by individual members of the profession. It is rather, in a larger sense, the spontaneous manifestation of a sentiment which "came into the air" because the time was ripe for it. Dimly defined it still remains, and yet it reveals in its faintly drawn outlines the embodiment of the idea that dentistry in the future is to include in its domain a large part of the field of general medicine. It also brings with it a suggestion that nothing requisite to the full, liberal equipment of the dental student will need to be sought for outside of the walls of the dental college.

The movement has, indeed, already commenced, as indicated by the extension of the dental college course. This practical step has been taken in recognition of the fact that the resolution adopted by the Medical Congress, at Washington, was a frank admission of ability on the part of the dental schools to fully qualify their students as representatives of the dental specialty of medicine. There is nothing in that resolution indicating a wish that dental students should pursue a course in the medical colleges. As a matter of fact it has been once or twice intimated that members of the medical faculty have betrayed indications of distrust of dental students who have applied for admission to medical colleges, avowing their purpose to practice dentistry.

If there be any truth in the statement it would seem to indicate doubt as to their assiduously applying themselves to subjects remote from dentistry so as to do credit to their alma mater. It may, however, have been merely disapproval of a practicing dentist devoting only part of his time to a course of study calculated to occupy *all* of the student's time.

It is not the purpose of this paper to discuss the question of the competency of dental colleges to educate their students in a three years' course to a point which shall fulfil and satisfy the expectations of the most exacting. The most conservative among the opponents of this recent movement extending the course of dental instruction will hardly deny that the dentist should know how to prescribe for his patients; should be able, at least, to identify all oral diseases, tumors, ranula, necrosis, salivary fistulæ, ozæna, the apthæ, and other diseases requiring medical attention, even though he be unable to treat these conditions. Such knowledge, at least on the dentist's part, he is required imperatively to possess. From this it is easy and natural to go a step further, and say that he needs to know more of reflex nervous re-action, diathesis, idiosyncrasies, shock, hysteria, febrile conditions, symptomatology, and so on.

It will be seen that in pursuing these and kindred subjects, indispensable to the dentist's education, the student will naturally be led, by processes of induction in his investigations, into fields remote from practical dentistry and trenching upon the domain of general medicine. Now the question arises when, and at exactly what point will he have gathered all the knowledge collateral to the subject of dentistry which he is likely to require in practice?

The resolution of the Medical Congress, to which we have referred, conveys a suggestion, by implication, that something may be omitted from the dentist's knowledge of medicine, his acquirements in his own special field of dentistry being accepted in lieu thereof. If this be a correct interpretation of the wording of that resolution, how are we to account for so extraordinary a concession by the medical faculty to the dental profession? No other class of medical specialists have been so favored. Was it not done in recognition of the fact that dentists, as a class, render to their patients unique service, requiring for its full and satisfactory performance less of the general knowledge of medicine than pertains to the ministrations of any other class of specialists?

It seems probable that in this view of the case the medical profession in a pure spirit of complaisance made this concession to dentistry. They recognized dentistry as a specialty of medicine. To have refused it such recognition would have been merely to deny to dentists the use of a convenient term. For that dentistry *is* a branch of the healing art is patent on the face of the statement; it is not a matter of opinion at all, it is a matter of fact.

Now, considering this question logically, the medical profession might have said to the dentists, we find you practicing *irregularly* a special branch of the healing art, which art, we as physicians profess to practice in its entirety. For the reason that you are not as we are, regularly educated physicians, we cannot recognize you as medical specialists. It is nothing to us that you find it inconvenient, or even impracticable to spend three years studying medicine, and three more years in the study of dentistry. The question which concerns us as physicians is, are you educated in medicine as are all our other specialists?

But what the medical profession did virtually say to the dentists is this: You dentists deserve special credit and honor for having created and developed a specialty from which we all these years have stood aloof. We admit you might with propriety question whether we, as physicians, could or would have tilled this field if you had never occupied it. We have never prepared any of our own members by our own unassisted efforts for this specialty. With all our knowledge of general and special surgery we have not even learned to extract teeth skillfully. We have been under the necessity of inquiring of you why teeth without living nerves ache. We have resorted to you for assistance in our attempts to reduce anchylosis of the jaws, for special splints, for the relief of neuralgia, etc. In short, we find that you have developed a specialty by methods of induction which brought you within the realm of our general field. We think you might have made still more rapid progress if you had begun your researches and investigations on the basis of a liberal medical education. But even *that* may be an open question. Your specialty is peculiar both in its offices and in the training required, and we are willing to admit you to membership among us on the most liberal terms consistent with the preservation of our character as a profession.

Notwithstanding the fact that the dental profession has shown full appreciation of the honor conferred upon it, there is still a strong undercurrent of sentiment cropping out occasionally in the inquiry whether a medical training, such as was evidently contemplated in the resolution, is best for the dentist. Men who raise this inquiry boldly proclaim that dentistry is essentially mechanical in its leading features, that it is in no important sense allied to medicine, and that experience shows that, with but few exceptions, members of the dental profession who have come into it from the ranks of the medical profession have not attained the highest rank. They profess to find in our dental schools, as now constituted, all requisite aids for a successful career in dental practice.

A separate essay might be written on the question of the mental characteristics of men who incline to dentistry, and others who incline to medicine. Admitting for the sake of argument that the highest order of mechanical talent is requisite for the attainment of a prominent rank in dentistry, the question arises, are men of this mental cast capable of pursuing profitably a course of study dealing so largely in theory, as the science of medicine? It cannot be too emphatically stated, that inasmuch as this question of a medical education for dentists must be considered, as regards its application to the collective body of coming dentists, we need to avoid a possible error, in considering only the comparatively small number (alleged to be) of successful medical-dentists, and, on our judgment of them, requiring of all future dentists a standard of attainments equal to theirs.

The writer's own individual opinion is, that the combination of the highest order of mechanical talent with such mental qualities as enable a man to excel as a physician is rarely found. It is, of course, impossible to say what progress in medical science might have been made, early in life, by some of the men now ripe in years, who enjoy a world-wide reputation as dentists. But if there be any significance in the fact that certain of these men, distinguished, as some one has put it, for being "all dentist," seldom or ever take noticeable interest in anything not directly pertaining to the *art* of dentistry; it must be admitted that they would probably be found wanting in the faculty of applying themselves to books and to study. Still, in all that we have just said, it may be objected that this combination of the very highest order of mechanical talent and the medical predilection, is not to be expected of the mass of students Many men in medicine and dentistry respectively, who never attain to more than local reputaation, nevertheless serve their patrons acceptably and enjoy their confidence. In answer to that objection we need only say that there is nothing unfair in comparing two representative members of these two professions, dentistry and medicine, selected as types of the highest attainment in their respective fields. It is only by measuring and comparing, each with the other, men who represent the very best products of their respective courses of training, that we shall be enabled to even approximate a decision as to what should be the average course to pursue in order to produce a new type of professional man, combining in himself the best qualities of these two distinct types.

It is not the object of this paper to consider what may be done by individual members of the dental profession already in practice to improve themselves in knowledge. It is not to our purpose to suggest that they enter upon a medical course. Until the present generation of dentists shall have passed away it will probably continue, as now, to be a mooted question whether dentists should be imperatively required to extend the range of their ministrations to their patients, beyond the care of their teeth. At the same time this very question is of interest and significance, since upon its final settlement, if such a thing were possible, might be predicated a feasible course to be pursued in the future instruction of dental students.

If it should finally be decided that students must study medicine before entering the dental college, would not consistency require some changes in the administration of the dental colleges? Shall the full-fledged medical graduate be required to sit for instruction at the feet of a corps of professors, some of whom are not medical graduates? The same dilemma would present itself in lengthening the course of the dental schools so as to embrace a partial medical course, unless it be that the instruction in the extended course were committed solely to the medical members of the dental faculty, in each particular school. But, even then, it seems that the graduate under the new dispensation must appear superior to some of his teachers. Even should the teacher have previously prepared himself by private study to escape from this foreseen dilemma, he might not find himself ranked on an equal footing with his pupil. This matter must, however, be faced boldly and frankly. Already some of the colleges, one or two, are requiring a preliminary examination in algebra, geometry, Latin, and other branches just beyond the verge of a common school education. It would be interesting to know how many teachers in dental schools have never studied these branches. This is not written in a spirit of cavil, but as a reminder that if a reform is to be instituted, no man should shrink from a personal fulfillment of all its requirements. Nothing is to be gained by mere pretentious claims. We are tempted to question the expediency of requiring a smattering of Latin, geometry, and history, of matriculates. A full course in Latin and Greek, extending over three or four years, is of important advantage to the student of anatomy, physiology, histology, therapeutics, and other kindred branches. But a mere introduction to these languages, which gives one no express familiarity with their root-origins, idiom, and structural peculiarities, is of no practical value. We see no significance whatever in this preliminary requirement, unless it be the demanding of a guarantee of the student's having acquired a habit of study, such as will enable him to grasp and appropriate the substance of the lectures he will listen to.

Even in that view of the matter it is a question whether previous devotion to book-study is the best kind of preparation for the listener to the spoken words of the lecturer. The study of books develops the reflective faculties; but it tends to divert the mind from a habit of quick and ready observation of facts presented in rapid succession by the speaker. The great requisite for the hearer is a hab't of attention and observation. This faculty is possessed in an eminent degree by reporters, who at their best seldom possess the peculiar habits of mental abstraction and deep reflection, such as mark the able editor. There seems to be some distinct recognition of these differing mental habits in the fact that some lecturers actually discourage note-taking while they are lecturing.

It is obvious, then, that the student who is likely to make the most substantial progress, under the present methods of instruction in the dental colleges, will be found to unite in his mental constitution the following faculties, viz., the habit of attention, quick apprehension, and the habit of reflecting.

It remains to be considered whether in a preliminary examination of a candidate for matriculation, natural aptitude is not of much greater importance than the mere question of so much Latin, geometry, history, etc., previously acquired. This query is suggested by the well attested fact that students in our dental and medical colleges, who possess much natural intelligence with but little book-learning, frequently pass the best examinations. And then follows another well attested fact, that these very prize-winners frequently fail in the practice of their professions.

So then we may well doubt whether the present means suggested, and in some cases actually in force, for anticipating incompetency in would-be matriculates, shall prove effective. And yet it is dificult to suggest any better course.

It may be necessary, before the desired object is attained, to revise some of our present ideas of conducting a course of instruction in our colleges. More study of books and fewer lectures may lead to the solution of the question.

In the foreign universities we find evidence of considerable latitude of opinion as regards the question of utility of the lecture system. The great requisite is ability on the student's part to pass his examination; the means of preparation being held virtually of secondary importance. A lecturer in one of our

[Cincinnati] medical colleges once answered this question: What is the important advantage of attending a medical course in college over a thorough course of reading under the direction of an intelligent preceptor? by declaring that it was in the opportunities for observation and practical demonstration in the hospitals. Is there not in this a hint for dental instructors? If we are to demand of the student better qualifications for study, shall he not be allowed more time for study, more practical demonstration, and less attention to lectures?

But whatever is to be the future education of dentists, the prime object, that of making students thoroughly competent *as dentists*, must not be lost to sight. The dentist came into being in response to a popular demand of a rather definite character, and a dentist he must be in order to meet that demand.

Possibly in the end this question will be solved independently of individual preferences, resolutions of medical congresses, or the aspirations of ambitious teachers. Neither the pride of the dentist who has enjoyed the advantage of a full medical course, nor the chagrin of the dentist who has missed that advantage, is certain materially to affect the result.

It is, after all, strictly a question of demand and supply. The man who best succeeds in saving teeth, who is most skillful in extracting teeth past saving, who shows superior judgment and artistic taste in providing artificial dentures, is certain to survive, by favor of public sentiment.

---

LISTER says, in the absence of chemical antiseptics, use boiling water, perfect cleanliness, wire, horsehair, or silkworm-gut sutures, and dry dressings. He thinks iodoform may not act directly on the bacteria, but induce chemical changes in their toxic products.

---

A LAW in Germany requires that all drugs intended for internal use be henceforth put up in round bottles, and those for external use be placed in hexagonal bottles. This enactment is precautionary against poisoning.

